# Estimating Efficacy of Indigenous Isolates of Three *Trichoderma* Species as Biocontrol Agents Against *Alternaria alternata* and *Curvularia spicifera*

**DOI:** 10.3390/jof12060421

**Published:** 2026-06-10

**Authors:** Lobna Hajji-Hedfi, Laith Khalil Tawfeeq Al-Ani, Takwa Wannassi, Amira Khlif, Boulbaba L’taief, Mavis Agyeiwaa Acheampong

**Affiliations:** 1 Regional Centre of Agricultural Research of Sidi Bouzid, Gafsa Road Km 6, P.O. Box 357, Sidi Bouzid 9100, Tunisia; 2 Laboratory of Agriculture Production Systems and Sustainable Development (LR03AGR02), Department of Agricultural Production, Higher School of Agriculture of Mograne, University of Carthage, Mograne Zaghouan, Tunis 1121, Tunisia; 3 School of Biology Science, Universiti Sains Malaysia, Minden 11800, Pulau Pinang, Malaysia; 4 Department of Plant Protection, University of Baghdad, Baghdad 10071, Iraq; 5 Biology Department, College of Sciences in Abha, King Khalid University, P.O. Box 960, Abha 62223, Saudi Arabia; 6 Department of Crop Science, University of Ghana, Legon, Accra P.O. Box LG 44, Ghana

**Keywords:** *Alternaria alternata*, biocontrol agent, *Curvularia spicifera*, plant-growth-promoting, post-harvest disease, *Trichoderma* spp.

## Abstract

Tomato is susceptible to various fungal pathogens, including *Alternaria alternata* and *Curvularia spicifera*, which can cause extensive post-harvest losses. Chemical fungicides have limited effectiveness in controlling post-harvest fungal pathogens and pose risk to human health and the environment. Therefore, this study assessed indigenous isolates of three species of *Trichoderma* (Tr1: *T. longibrachiatum*; Tr2: *T. harzianum*; and Tr3: *T. asperellum*) as biocontrol agents against two fungal pathogens in vitro and in vivo and determined their physicochemical analysis and plant-growth-promoting traits. The three species of *Trichoderma* exhibited catalase production in vitro, while *T. longibrachiatum* and *T. asperellum* showed the highest potential for plant-growth promotion by producing indole-3-acetic acid and phosphate solubilization but not nitrogen-fixing capability. *T. harzianum* showed lower potential in these traits. Mycelial growth was found to be maximum (5.77–12.27 cm) at 30 °C and a pH of 7–9, but inhibition (2.60–5.13 cm) was recorded at the highest temperature (45 °C) and pH (11). In vivo, studies on tomato fruits indicated that *T. longibrachiatum* and *T. asperellum* significantly (*p* < 0.05) reduced lesion diameters of *A. alternata* by 53.60% and 48.71%, respectively, and *C. spicifera* by 55.58% and 56.19%, respectively, relative to the infected control. Besides their antifungal efficacy, the three species of *Trichoderma* enhanced tomato seedling growth, particularly at 1/10 filtrate dilution, and improved fruit quality parameters by increasing firmness and nitrate content, while reducing oxidative stress. Physicochemical analysis indicated that *Trichoderma*-treated fruits had better firmness, pH, and nitrate value coupled with a reduction in oxidative stress (reduced malondialdehyde content) compared to pathogen-infected controls. The indigenous isolates of the three species of *Trichoderma* provided high efficacy as biocontrol agents of the two fungal pathogens that cause post-harvest losses of tomato, suggesting that biological control can replace synthetic chemicals in preserving tomato under storage conditions and contribute to agricultural sustainability.

## 1. Introduction

Tomato (*Solanum lycopersicum*) is among the world’s most economically valuable vegetable crops [1]; however, its production is significantly hampered by fungal pathogens that result in devastating pre- and post-harvest diseases. Globally, tomato production exceeded 190 million tons in 2024, highlighting its enormous economic and nutritional importance as a key vegetable contributing to food security, dietary antioxidants, and human health [2]. Plant pathogenic fungi represent major barrier to tomato production and pre-harvest quality. Most notable among these are *Alternaria alternata* [3], the causative agent of early blight, and *Curvularia spicifera*, which is linked with fruit rot, resulting in enormous yield losses, and adversely affecting marketability and nutritional quality [4]. Such fungal diseases not only cause quantitative yield reductions but also qualitative deterioration of fruits during storage and transport, threatening both producer income and consumer safety [5].

Conventional management strategies, particularly using synthetic fungicides, have come under scrutiny due to their toxicity to the environment, potential health effects, and the buildup of pathogen resistance [6]. Although cultural and physical control measures such as crop rotation, sanitation, and storage-temperature regulation can mitigate disease severity, they are often insufficient alone under high pathogen pressure [7].

The use of fungicides also leads to residue accumulation and ecological imbalance, which therefore demand safer and more sustainable and environmentally friendly alternative solutions, with biological control agents such as *Trichoderma* species regarded as prime candidates [8]. *Trichoderma* species are well documented for being dual-functional both as biocontrol agents and as plant-growth-promoting fungi, with the ability to suppress phytopathogens by competition, antibiosis, and induced systemic resistance [9,10]. Additionally, *Trichoderma* spp. improves plant health by solubilizing nutrients and producing phytohormones [9,10]. Recent work revealed that *T. harzianum* T34 induced host-specific gene expression and enhanced defense enzyme activity in tomato, demonstrating molecular evidence of systemic resistance. Consequently, transcriptomic analysis of tomato plants primed with *T. harzianum* T34 identified 1786 significantly differentially expressed genes (DEGs) and a core set of 156 genes consistently affected by priming, including upregulation of defense-related transcription factors, genes associated with secondary-metabolite and oxidative-stress pathways, indicating systemic transcriptional reprogramming after *Trichoderma* treatment [11]. Moreover, antifungal metabolites from *Trichoderma reesei* (T1) significantly reduced gray mold (*Botrytis cinerea*) incidence in post-harvest tomato to as low as 8.6%, confirming the potential of secondary metabolites for effective biological control. Furthermore, *Trichoderma* treatment also stimulated antioxidants (increased total phenolic, flavonoid contents), and delayed senescence in tomato fruits through defense enzyme activities (peroxidase, phenylalanine ammonia-lyase, and polyphenol oxidase) [12]. Accordingly, pre- and post-harvest studies, with salicylic acids improved antioxidant systems and extended the shelf life of tomato [13,14]. This pattern supports biological control, in which *Trichoderma* suppresses pathogens and increases fruit resistance [5]. Nonetheless, even with these established advantages, the manner through which *Trichoderma* spp. are inhibitory to post-harvest pathogens such as *A. alternata* and *C. spicifera* remain not well researched, particularly in relation to their enzymatic activity and environmental tolerance [3,10,15]. Further, there is still a lack of studies combining both the post-harvest biocontrol efficacy and plant-growth-promoting traits of *Trichoderma* spp. against major tomato pathogens such as *A alternata* and *C. spicifera*. As much as their potential has been demonstrated under controlled laboratory conditions, field applications require precise information on optimal growth conditions to maximize their performance [16]. Additionally, the ability of *Trichoderma* to produce extracellular enzymes (e.g., catalase, protease, β-1,3-glucanase) and plant-growth-promoting metabolites (e.g., indole-3-acetic acid, hydrocyanic acid) varies greatly among species, yet comparative studies on these traits are scarce [9]. Furthermore, while the antagonism of *Trichoderma* against soil-borne pathogens is well documented, its post-harvest application in preserving tomato fruit quality particularly from a physicochemical and biochemical perspective has been scarcely investigated. It is essential to fill these gaps in knowledge to develop targeted biocontrol strategies that can be included in sustainable agriculture [8].

Thus, this study aimed to: (i) assess the in vitro plant-growth-promoting and enzymatic activities of three species of *Trichoderma* (*T. longibrachiatum*, *T. harzianum*, and *T. asperellum*); (ii) determine the effect of temperature and medium pH on the growth of mycelia, and (iii) analyze their in vivo effectiveness against *A. alternata* and *C. spicifera* diseases on tomato fruits with particular reference to pathological, physicochemical, and biochemical responses. This study expands the research on these local isolates of *Trichoderma* by providing new insights into their efficacy against post-harvest tomato pathogens, supporting their use in sustainable disease management strategies.

## 2. Materials and Methods

### 2.1. Fungal Species

The present study used three previously characterized *Trichoderma* isolates, Tr1 (*T. longibrachiatum*), Tr2 (*T. harzianum*), and Tr3 (*T. asperellum*), with the GenBank accession numbers OP799680, OP799678, and OP799679, respectively. These isolates were obtained from the Plant Protection and Biological Sciences Laboratory at the Regional Centre of Agricultural Research (CRRA) of Sidi Bouzid, Tunisia. These species of *Trichoderma* were isolated from the rhizosphere of tomato plants and selected as local isolates with biocontrol efficacy against fungal pathogens of tomato, and with plant-growth-promoting potential, as previously reported by [17,18]. The isolates were identified based on morphological criteria and confirmed by molecular identification [18,19]. *A. alternata* (GenBank accession number: PQ892125) and *C. spicifera* (GenBank accession number: PQ892128) were isolated from naturally infected tomato fruits (cv. Firenze) exhibiting symptoms of early blight and fruit rot, respectively.

### 2.2. In Vitro Plant-Growth-Promoting Traits and Extracellular Enzymes

The plant-growth-promotion ability of the three species of *Trichoderma* was assessed via their ability to synthesize indole-3-acetic acid (IAA), solubilize phosphate (P), fix atmospheric nitrogen (N), and produce hydrocyanic acid (HCN). The study also investigated whether the *Trichoderma* species can produce a group of extracellular enzymes such as catalase (Cat), pectinase (Pec), protease (Pro), and β1,3-glucanase (Glu) to provide their possible mechanism of action towards plant-growth promotion.

IAA: The ability of the tested three species of *Trichoderma* to produce IAA was assayed using a modified qualitative colorimetric assay from the method of [20]. One fungal agar plug was used to inoculate potato dextrose agar (PDA) medium and incubated at 28 °C for 48 h. Following incubation, an untreated Whatman filter paper disk of 5 cm in diameter, pre-soaked in Salkowski’s reagent, was placed on the fungal culture. IAA production was indicated by a color change of the filter paper from yellow to pinkish brown, resulting from the reaction between Salkowski’s reagent and IAA produced by the fungus [20].

P: Phosphate solubilization by the tested species of *Trichoderma* was assessed by culturing the fungi on Pikovskaya’s medium at 28 °C for 7 d. The formation of a clear halo surrounding the fungal growth served as an indicator of phosphate solubilization [21].

N: Nitrogen-fixation capacity of the tested species was assayed in Norris Glucose Nitrogen-Free (N-free) medium. The capacity of the isolates to utilize atmospheric nitrogen as a nitrogen source was established by growing them on N-free medium, which would be an indication of nitrogen fixation. After 5 d of incubation at 30 °C, the growth in the form of a film on the surface of the medium indicated the nitrogen-fixing capability of the isolate [21].

HCN: The potential of the tested species to produce HCN was evaluated with a chromogenic assay. Fifteen milliliters of PDA supplemented with 4.4 g L^−1^ glycine was added to Petri plates, and upon solidification, a 0.5 cm diameter fungal agar plug was placed on the agar surface. A Whatman No. 1 filter paper disk, wetted in an alkaline picrate solution (prepared from 2.5 g picric acid and 12.5 g Na_2_CO_3_ in 1 L of distilled water, pH 13), was fixed to the inner lid of each Petri plate. The plates were incubated at 28 ± 2 °C for 4 d. HCN production was indicated by a change in the color of the filter paper, ranging from weak (orange red to yellow), moderate (brown), to strong (reddish-brown) [22].

Cat: Catalase activity of the tested species was assayed using the method of [22]. A small drop of H_2_O_2_ solution was mixed with a sample of the fungal colony on a microscope slide. The prompt appearance of visible gas bubbles, indicating oxygen release, was a confirmation of catalase activity, demonstrating the degradation of H_2_O_2_ by the enzyme.

Pec: Pectinase activity among the tested species was quantified in a zone-formation assay according to the protocol described by [23]. This protocol used agar plates solidified with pectin as substrate in order to detect pectinase activity, a marker of the ability of the fungus to break down pectin, a major part of the plant cell wall. The pectin agar was inoculated with a fungal plug and left at 28 °C for 5 d. The plates were flooded with a 0.05% (*w*/*v*) ruthenium red solution, a specific stain for pectin which stains the medium red. The free ruthenium red was then removed from the plate by rinsing with distilled water. The appearance of a transparent halo around the fungal colony after rinsing was an indicator of pectinase production, as this was the area where the pectin had been broken down by the secreted fungal enzymes. This halo development points to the enzymatic potential of the fungus and suggests their potential role in maceration of plant tissue, thereby facilitating the acquisition of nutrients from the broken-down plant material [23].

Pro: *Trichoderma* species were screened for protease activity by a zone-formation assay on skim milk agar, which is a medium containing casein, a milk protein substrate for proteases [24]. The fungal plug was inoculated onto the solidified skim milk agar plates and incubated at 28 °C for 5 d. The plates were observed after incubation for the presence of a clear halo around the fungal colonies. The appearance of this clear zone was a sign of protease production, depicting the area in which the casein had been hydrolyzed by the fungal proteases secreted. The enzymatic activity observed shows the potential of the fungus to break down proteins, an activity with uses across many disciplines [24].

Glu: Agar plates gelled with laminarin (1 g/L) as the substrate of β-1,3-glucan, peptone (0.5 g/L), and yeast extract (0.1 g/L) for the support of fungal growth were inoculated with a plug of the fungus and cultured at 28 °C for 5 d. Production of β-1,3-glucanase was indicated by the development of a clear halo region surrounding the fungal colony, the region where laminarin had been hydrolyzed by the diffused enzyme. Absence of stainable substrate in this region facilitated visualization, and the size of the clear zone correlated with the strength of β-1,3-glucanase activity secreted by the fungus [21]. For each parameter, five replicates of each assay were conducted, and the assays were repeated three times to ensure that the results were repeatable.

### 2.3. Physiological Properties of the Tested Trichoderma Species

The growth of the tested three species of *Trichoderma* was assessed at a temperature range comprising the minimum, optimum, and maximum. A disk of 5 mm in diameter of each fungus species (four days old) was centrally inoculated on PDA plates. Mycelial growth was determined daily in the same hour each day for 7 d (day 1, day 2, day 3, day 4, day 5, day 6, and day 7) by measuring the average of two perpendicular diameters of each colony. Measurements were taken at the same marked positions on each plate daily to ensure consistency. Experiments were conducted at 19, 30, and 45 °C in three replications (five Petri plates per isolate for each repetition) for each temperature tested [25]. Furthermore, the optimal pH for growth of the tested *Trichoderma* species was assessed by preparing 100 mL of PDA medium in 250 mL Erlenmeyer flasks, adjusting the pH to 5, 7, 9, and 11 using 0.1 N HCl or NaOH as determined with a digital pH meter. A disk of 5 mm in diameter of each fungal species (4-day-old culture) was centrally inoculated on the PDA plates. Growth of mycelium was measured daily for 8 d, with three replicates of five plates of each treatment at 25 °C [25]. No separate control treatment was included; instead, growth at each temperature and pH was compared directly to determine the optimal conditions.

### 2.4. In Vitro Antagonistic Activity of the Tested Trichoderma Species Against Alternaria alternata and Curvularia spicifera

The three species of *Trichoderma* were evaluated for their potential antagonistic activity against tomato pathogens *A. alternata* and *C. spicifera* using a dual-culture assay method with three replicates (five plates of each treatment per replicate) as indicated above. Control plates were inoculated with the pathogens alone under the same conditions. A 5 mm disk of each fungal pathogen of a seven-day-old culture was placed onto one side of a 9 cm Petri plate of PDA medium (10 mL), and on the opposite side of the plates, a 5 mm plug of the antagonistic *Trichoderma* isolate was placed at 3 cm from the pathogen mycelium. The mycelial growth was measured daily in mm for 7 d following [9]. The inhibition percentage was calculated using the formula:

(1) $$\mathrm{Inhibition}\;\mathrm{percentage}\;(\%)\;=\;\frac{L-I}{L}\times 100$$

where L is the radial growth of fungi in control and I is the radial growth of fungi in the presence of the tested *Trichoderma* isolate.

Additionally, the area under the disease progress curve (AUDPC) was assessed to compare the overall efficacy of applied treatments in inhibiting fungal pathogen growth over time. This metric integrates repeated measurements of mycelial expansion (cm) into a single value (cm·days) using the trapezoidal method described by [26] with the formula below (2):

(2) $$AUDPC=\sum_{i=1}^{n-1}(\frac{yi+yi+1}{2})\;\times (ti+1-ti)$$

where *yi* = mycelial growth (cm) at the *i* observation time; *ti*: time (days) at the *i* observation time; and *n*: total number of observations.

### 2.5. Efficacy of Trichoderma Fungal Suspension on Tomato Seedlings Growth

Tomato seeds of the cultivar Firenze obtained from the Plant Protection Laboratory of CRRA were washed three times with ethanol, rinsed with sterile distilled water and allowed to dry at room temperature. The tested *Trichoderma* species were cultured in PDA medium for 4 d at 28 °C, and 5 mm diameter mycelial plugs of each species were aseptically collected from the growing cultures and inoculated into Erlenmeyer flasks separately containing potato dextrose broth (PDB), and incubated with agitation for 4 d in a rotary shaker incubator (Optic Ivymen System, Barcelona, Spain) at 150 rpm and 28 °C. After removing the mycelial pellets using 0.45 μm pore size filters, each fungal cultrate was refiltered using a 0.22 μm membrane filter. Following cultures filtration, the filtrates were subsequently diluted with sterile distilled water to obtain doses of 0, 1/10, 1/50, 1/100, 1//200, and 1/500. Seeds were immersed in 50 mL of each suspension for 30 min. For the negative control, seeds were immersed in sterile distilled water without *Trichoderma* species filtrates under the same conditions. Ten repetitions of each treatment were carried out for each dilution, and the experiment was repeated three times. Seven days post-treatment, hypocotyl and radicle (seedling) lengths were measured to calculate the vigor index (VI) of the seedling according to the formula:

(3) VI = Percentage of germination × (length of the hypocotyl + length of the radicle)

### 2.6. In Vivo Evaluation of Trichoderma Fungal Suspension Biocontrol Effect on Tomato Fruits Inoculated with Alternaria alternata and Curvularia spicifera

In order to compare the efficacy of the various *Trichoderma* species in controlling *A. alternata* and *C. spicifera* infection on tomato fruits, a controlled experiment was conducted on matured tomato fruits at the commercial red-ripe stage. Uniform-sized ripe fruits of the ‘Firenze’ cultivar harvested from a biological Sidi Bouzid field, Tunisia, which were free from visible injuries or disease were selected manually. A randomized complete block design with three blocks, each with ten containers, was used and the entire experiment was replicated twice for data reliability [10]. Six treatments were tested: T1: negative control (healthy fruits); T2: infected control (fruits treated only with *A. alternata* or *C. spicifera*); T3: Tr1 culture filtrate + phytopathogen (*A. alternata* or *C. spicifera*); T4: Tr2 culture filtrate + phytopathogen; T5: Tr3 culture filtrate + phytopathogen; and T6: SA + phytopathogen. Fruits were prepared by surface sterilization in 2.5% NaClO, followed by washing in sterile water and air-drying under laminar flow hood before imposing a standard 5 mm deep wound at the blossom end. Subsequently, 20 μL of undiluted filtrate of each *Trichoderma* suspension (10^6^ spores/mL) or 20 μL of salicylic acid (SA) (C_6_H_4_(OH)CO_2_H, 0.001 M) was spread on the wound sites and then incubated for 2 h prior to being inoculated with 20 μL of the phytopathogen spore suspension (10^6^ spores/mL) for all treatments with the exception of the negative control (fruits treated only with 20 μL of distilled water). The treated fruits were then stored in humidified plastic containers on sterile wet paper and covered in plastic bags to maintain more than 90% humidity. The treatments were incubated for 7 d in a growth chamber at 21 °C with a 8:16 h (day:night) photoperiod. Each pathogen was tested independently. All the treatments described above were applied separately either with *A. alternata* or with *C. spicifera*, and no combined inoculation of both pathogens was performed in the same fruit.

The pathological (rot diameter), morphometric (firmness), physicochemical (pH, titratable acidity, sugar content, nitrate content, and electrical conductivity), and biochemical (total phenolic content, malondialdehyde, and total protein content) attributes were measured 7 d after treatments to determine the antifungal activity of the above-mentioned treatments on tomato fruits [18,19].

### 2.7. Statistical Analysis

A one-way analysis of variance (ANOVA) was conducted using R software version 4.5.1 to evaluate the presence of significant differences among the treatment groups. Before proceeding with the ANOVA, the means of replicating samples were calculated to assess data consistency. All independent repetitions of the experiment yielded highly consistent results, showing identical trends across all evaluated parameters in this study. To ensure the validity of the ANOVA results, the assumptions of normality and homogeneity of variances were verified according to the Shapiro–Wilk test and Levene’s test. Duncan’s Multiple Range Test was subsequently applied as a post hoc analysis to pinpoint specific treatment groups that exhibited significant differences (*p* ≤ 0.05) in their means. Data was expressed as mean ± standard error (SE). Diagram visualizations were created using the “ggplot2” package in RStudio (Version 2025.05.1+513).

## 3. Results

### 3.1. Plant-Growth-Promoting and Extracellular Enzymes Produced by the Trichoderma Species

Table 1 illustrates a comparison among the three species of *Trichoderma* based on extracellular enzyme activities and plant-growth-promoting traits. Catalase was produced by all the three species of *Trichoderma*. However, none of the three species produced pectinase, protease, amylase, and β-1,3-glucanase. In terms of PGP activities, *T. longibrachiatum* and *T. asperellum* both exhibited the highest PGP profile with respect to the production of indole-3-acetic acid, hydrocyanic acid, and phosphate solubilization. On the other hand, *T. harzianum* produced hydrocyanic acid and solubilized phosphate. None of the three species fixed atmospheric nitrogen.

### 3.2. Temperature and pH Conditions Modulate Trichoderma Species Mycelial Growth

At 19 °C, all three *Trichoderma* species demonstrated gradual mycelial growth over the seven days, with significant differences observed between the species. *T. longibrachiatum* exhibited the lowest growth rate, starting with 0.07 cm on day 1 and reaching 3 cm by day 7. *T. harzianum* showed a higher growth rate, starting with 0.17 cm and reaching 5.6 cm by day 7. *T. asperellum* displayed a similar trend to *T. harzianum*, starting with 0.63 cm and reaching 5.13 cm by day 7. At 30 °C, all three species exhibited their highest growth rates. *T. longibrachiatum* grew from 1.7 to 7.63 cm, *T. harzianum* from 2.7 cm to 7.27 cm, and *T. asperellum* from 2.6 cm to 6.63 cm (at 1 and 7 d after incubation, respectively). The growth rates were statistically similar among the three species at 30 °C. At 45 °C, mycelial growth was significantly inhibited across all species. *T. longibrachiatum* showed a limited growth, starting at 0 cm and reaching only 2.6 cm by day 7. *T. harzianum* exhibited a similar trend, starting at 0 cm and reaching 3.37 cm. *T. asperellum* showed a slight growth, starting at 0.1 cm and reaching 4.17 cm. Overall, the data suggests that 30 °C is the optimal temperature for mycelial growth for all three *Trichoderma* species, while 19 °C allows for slower growth, and 45 °C severely inhibits growth (Table 2).

Mycelial growth was optimum for all three *Trichoderma* species in the medium adjusted to pH 7 and 9, with the highest growth rate recorded in *T. asperellum*. The mycelial growth of *T. longibrachiatum* increased with increasing pH of up to 9 and decreased at pH 11. At pH 5, growth was modest and steady throughout the seven days, rising progressively from 1.23 cm at D1 to 4.63 cm at D7. At pH 7, growth was slightly higher, with 5.77 cm at D7. The highest growth occurred at pH 9, where mycelial growth reached 8.7 cm on D7. At pH 11, growth was highly inhibited, particularly in the early growth stages, where very little growth was observed. Similar trends were recorded for *T. harzianum* with the highest growth rate at pH 7 and 9. At pH 5, the growth was steady, rising from 2.77 cm at D1 to 5.37 cm at D7. At pH 7, the growth was comparatively higher at 5.97 cm by D7. At pH 9, growth amounted to 7.1 cm at D7. At pH 11, growth was once more drastically inhibited, particularly at the later incubation stages, and only amounted to 4.3 cm at D7. *T. asperellum* grew faster, especially at pH 9, where the mycelial growth attained 12.27 cm at D7. The growth was also considerably better at pH 7, with 8.67 cm at D7. At pH 5, the growth was constant, from 1.9 cm on D1 to 5.17 cm at D7. At pH 11, the growth was significantly retarded, with just 4.43 cm at D7 (Table 3).

### 3.3. Mycelial Growth Inhibition of Trichoderma Species Against Alternaria alternata and Curvularia spicifera

**Figure 1:**
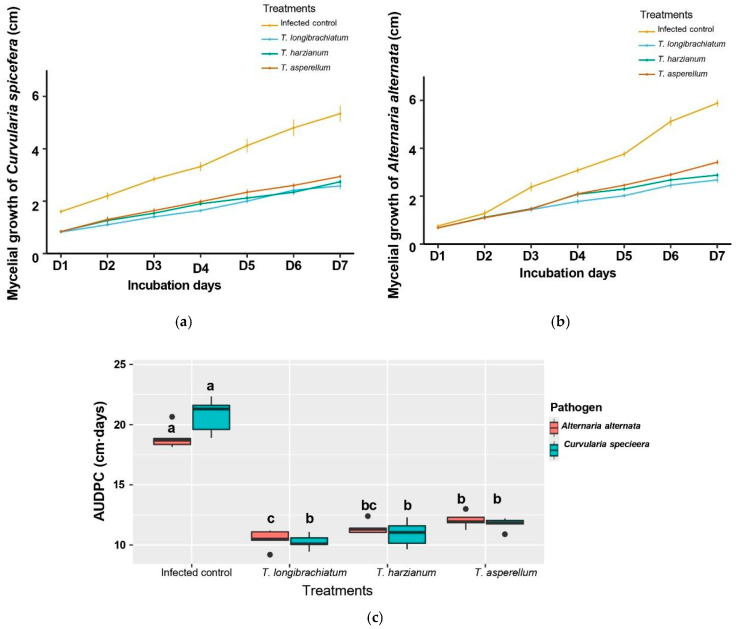
Temporal dynamics of mycelial growth of *Curvularia spicifera* (**a**) and *Alternaria alternata* (**b**) over seven days under direct confrontation and mean area under the disease progress curve (AUDPC) values (cm·days) (**c**) of mycelial growth of *Alternaria alternata* and *Curvularia specifera* as influenced by *Trichoderma* treatments (Tr1: *T. longibrachiatum*; Tr2: *T. harzianum*; and Tr3: *T*. *asperellum*). Mean values of each box followed by the same letter are not significantly different. C+: Infected control. Distinct letters above the boxplots indicate significant differences between treatments according to Duncan’s test (*p* ≤ 0.05). Five replicates, repeated in three independent experiments (n = 5 × 3).

**Figure 2:**
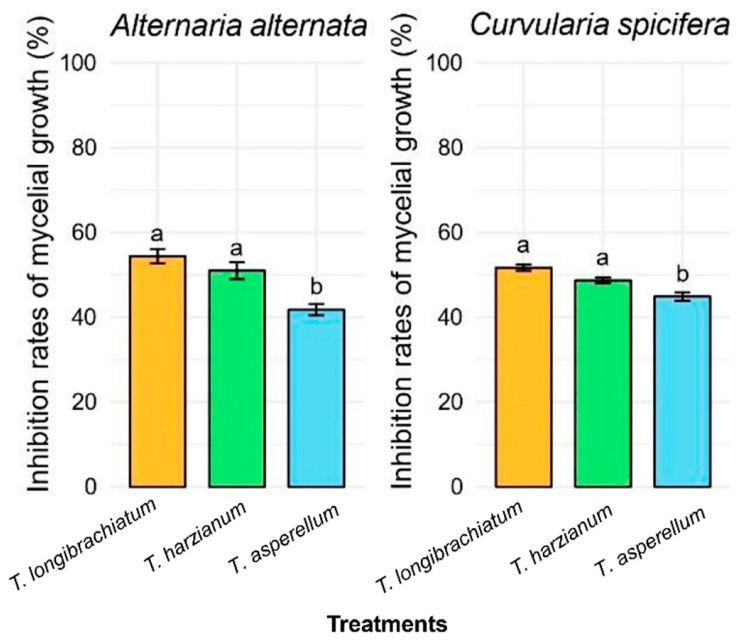
Inhibition rates of *Alternaria alternata* and *Curvularia spicifera* mycelial growth by the *Trichoderma* species treatments (Tr1: *T. longibrachiatum*; Tr2: *T. harzianum*; and Tr3: *T. asperellum*) in seven days of incubation relative to infected control. Distinct letters above the boxplots indicate significant differences between treatments according to Duncan’s test (*p* ≤ 0.05). The value represents the mean of the 5 replicates, repeated in three times (n = 5 × 3), ± standard error.

The antagonistic effects of three species of *Trichoderma* were consistently demonstrated across all evaluation methods, including mycelial growth, AUDPC analysis and rates of inhibition (Figure 1 and Figure S1). Temporal growth curves (Figure 1a,b) revealed that *T. longibrachiatum* (Tr1) exhibited the strongest suppression of both pathogens, reducing *C. spicifera* growth to 2.58 cm (51.7% inhibition) and *A. alternata* to 2.32 cm (51.8% inhibition) by day 7 compared to infected controls (5.34 cm and 4.81 cm, respectively). *T. harzianum* (Tr2) and *T. asperellum* (Tr3) showed progressively slightly lower inhibition than Tr1, with Tr2 achieving 48.7–49.2% suppression and Tr3 showing 44.9–45.3% reduction across both pathogens. These patterns were confirmed by AUDPC analysis (Figure 1c), where *T. longibrachiatum* yielded significantly lower values (*C. spicifera*: 10.2 cm·days; *A. alternata*: 8.9 cm·days) compared to controls (18.7 and 16.3 cm·days, respectively; *p* < 0.05); however, *T. harzianum* (Tr2) and *T. asperellum* (Tr3) showed slightly similar trends. Figure 2 visualization confirmed these findings, with Tr1 displaying the important inhibition zones (54.4% for *C. spicifera*; 51.0% for *A. alternata*), while Tr3 showed the lightest suppression (44.9% and 41.8%). Overall data showed that antagonist efficiency is ranked as Tr1 > Tr2 > Tr3 against both pathogens, which may suggest that of the studied isolates, Tr1 has the strongest and most consistent antifungal activity, whereas Tr3 is the least effective.

### 3.4. Trichoderma Species Culture Filtrates Stimulate Tomato Seedling Growth in a Dose-Dependent Manner

Tomato seeds were treated with different dilutions (0, 1/10, 1/50, 1/100, 1/200, and 1/500) of the *Trichoderma* species culture filtrates to investigate the best dilution for seedling growth. All dilutions were expressed as volume/volume (*v*/*v*) ratios of filtrate to sterile distilled water. Seven days following treatment, the length of the seedlings was measured and compared to the negative control treated with water only.

The ANOVA results revealed a significant difference in seedling growth among the applied treatments (*p* < 0.05) compared to the control.

**Figure 3:**
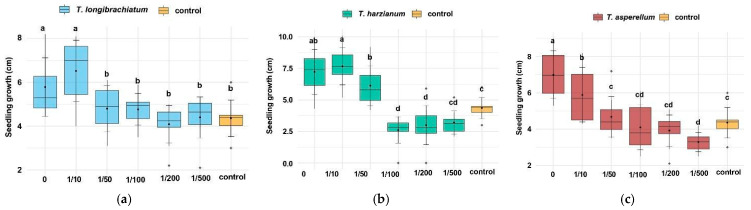
Effect of *Trichoderma* treatment (Tr1: *T. longibrachiatum* (**a**); Tr2: *T. harzianum* (**b**); and Tr3: *T. asperellum* (**c**)) filtrate dilutions at 0, 1/10, 1/50, 1/100, 1/200, and 1/500 on tomato seedling growth compared to control. Distinct letters above the boxplots indicate significant differences between treatments according to Duncan’s test (*p* ≤ 0.05). Each value represents the mean of 10 replicates, repeated in three independent experiments (n = 10 × 3), ± standard error.

For *T. longibrachiatum* (Tr1), the control produced moderate growth of 4.36 cm, while the 1/10 dilution showed the highest promotion (6.52 cm), followed by a clear reduction at higher dilutions progressively (1/100: 4.77 cm; 1/500: 4.4 cm) (Figure 3a and Figure S2). *T. harzianum* (Tr2) exhibited the highest seedling growth at 1/10 (7.67 cm), followed by zero dilution accounting for 7.23 cm, surpassing its treatment control (7.23 cm), after which growth declined at 1/50 to 1/500 dilutions (Figure 3b). For *T. asperellum* (Tr3), the undiluted filtrate yielded the best results (7.01 cm), followed by 1/10 (5.89 cm), with progressively decreased effects at higher dilutions (1/500: 3.29 cm). Across all tested isolates, the same tendency was observed, where the higher concentration of the undiluted treatment (1/10) exhibited the most relevant seedling growth apart from samples tested with *T. asperellum*, which may indicate a dose-dependent effect of *Trichoderma* species.

### 3.5. Biocontrol Potential of the Studied Trichoderma Species Against Alternaria alternata and Curvularia spicifera in Tomato Fruits

**Figure 4:**
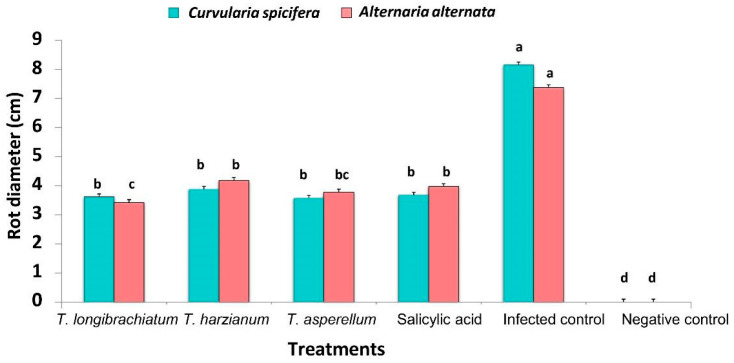
Effect of preventive treatments of *Trichoderma* species (Tr1: *T. longibrachiatum*; Tr2: *T. harzianum*; and Tr3: *T. asperellum*) and salicylic acid (SA) on the rot diameter of tomato fruits inoculated with *Alternaria alternata* and *Curvularia spicifera*. C+: infected control; C−: negative control. Distinct letters above the boxplots indicate significant differences between treatments according to Duncan’s test (*p* ≤ 0.05). Each value represents the mean of 10 replicates per block across three blocks, evaluated in two independent experimental repetitions (n = 10 × 3 × 2), ± standard error.

The rot diameter of both pathogens on infected fruits was quantified as an indicator of pathogenic impact to emphasize the extent of the damage. *T. longibrachiatum* exhibited the best efficacy in decreasing the rot diameter of *A. alternata*, with 3.42 cm recorded, followed by *T. asperellum* (3.78 cm), while salicylic acid exhibited a slightly similar protection effect (3.97 cm), all of which conferred good protection compared to the inoculated infected control (7.37 cm) (Figure 4 and Figure S3). *Trichoderma* species and salicylic acid resulted in significantly reduced rot diameters compared to the infected control (8.15 cm). *T. asperellum* showed the lowest rot diameter of *C. spicifera* (3.57 cm), followed by *T. longibrachiatum* (3.62 cm) and *T. harzianum* (3.88 cm). Salicylic acid resulted in a rot diameter of 3.68 cm. Figure 4 showed that the three species of *Trichoderma* significantly reduced fruit rot diameter caused by both pathogens compared to the infected control, with Tr1 and Tr3 exhibiting the strongest biocontrol effect. Although SA also reduced lesion size, the *Trichoderma* treatments showed comparatively greater suppression overall.

The preventive treatments against *A. alternata* and *C. spicifera* significantly changed the physicochemical properties of tomato fruits. *T*. *longibrachiatum* preserved favorable fruit quality, but pH responses were pathogen-dependent. Under *C. spicifera*, Tr1 showed the highest pH (4.40), indicating a less acidic condition, whereas under *A. alternata*, Tr1 exhibited an intermediate pH value (4.24), with other treatments such as Tr3, salicylic acid and the infected control presenting higher pH values (4.16–4.64). Tr1 measured the highest electrical conductivity (6.49 mS/cm) and maintained moderate conductivity under *A. alternata* (4.43 mS/cm), reflecting a better ionic regulation and metabolic stability. Tr1-treated fruits revealed the lowest sugar level (4.63 °Brix) for *C. spicifera*, while *A. alternata* exhibited the highest sugar level (6.47 °Brix), with a moderate titratable acidity in both pathogens, indicating a more balanced organic acid–carbohydrate metabolism compared to the infected control. *T. longibrachiatum* contained the highest nitrate content of 2400 µg/mL in *C. spicifera but* a lower nitrate of 1633 µg/mL in *A. alternata* with moderate firmness. In contrast, *T. harzianum* showed a lower pH (4.17 under *C. spicifera* and 4.1 under *A. alternata)*, which is more acidic than Tr1. It induced relatively high electrical conductivity (6.19 mS/cm) under *C. spicifera* infection, while lower values were for tomato infected with *A. alternata* of 4.35. For both of the pathogens, Tr2 treatment induced the highest sugar content with the lowest firmness, suggesting more rapid fruit softening. *T. asperellum* displayed contrasting electrical conductivity responses, showing the highest EC under *A. alternata* (6.17 mS/cm) but the lowest EC under *C. spicifera* (2.35 mS/cm), with moderate firmness values in both cases, which may indicate a pathogen-dependent stabilization response of ionic leakage. The infected control exhibited the poorest fruit quality overall, including a pH of 4.02, the lowest electrical conductivity (1.03 mS/cm), 6.47 °Brix sugar content, the lowest titratable acidity (4.37 g/10 mL), 2266.67 µg/mL nitrate content, and the lowest firmness (0.53). Meanwhile, the negative control represented the physiological optimum state (Table 4).

The obtained data showed significant differences among the treatments for each of the parameters measured (*p* < 0.01). For malondialdehyde (MDA), the lowest levels were recorded in *T. asperellum* under *A. alternata* infection (1.38 µmol/g), while under *C. spicifera* the lowest MDA was observed in *T. longibrachiatum* (1.86 µmol/g), indicating a pathogen-dependent oxidative stress alleviation.

Based on the overall protein concentration, salicylic acid ranked far above the rest, with an optimum value of 50.85 mg/g, indicating considerable protein accumulation. Furthermore, the total protein content showed no significant difference under *A. alternata* (*p* ≥ 0.05), while under *C. spicifera* infection *T. asperellum* exhibited the highest protein concentration (31.24 mg/g). Regarding phenolic compounds, *T. longibrachiatum* significantly enhanced the total phenolic content under *A. alternata* (1.11 µg/g), whereas under *C. spicifera* the negative control recorded the highest phenolic level (1.04 µg/g) (Table 5).

Focusing on malondialdehyde, the findings revealed significant differences between the treatments. This can be interpreted as the *Trichoderma* species, specifically *T. asperellum*, effectively alleviating oxidative stress, as evidenced by its low MDA level (1.38 µmol/g). On analysis of total protein content, despite numerical differences, the lack of statistical significance means these treatments did not significantly alter overall protein content (*p* ≥ 0.05). However, the analysis of total phenolic content showed that *T. longibrachiatum* significantly boosted the level of these antioxidant substances.

## 4. Discussion

In vitro assays of the studied *Trichoderma* species in the current study resulted in a range of plant-growth-promoting and enzymatic activities reflecting their multifunctional biocontrol activity. *T. longibrachiatum*, *T. harzianum*, and *T. asperellum* produced catalase, a key enzyme that detoxifies reactive oxygen species and minimizes oxidative stress in plants. This enzymatic activity is especially relevant in post-harvest uses, where fungal infection commonly results in oxidative stress in host tissue [27]. *T. asperellum* and *T. longibrachiatum* were found to have the potential for indole-3-acetic acid production, a plant phytohormone that promotes root growth and facilitates nutrient absorption in plants [28]. IAA production indicates that apart from possibly playing a role in disease suppression, *Trichoderma* species can also play a role in enhanced plant vigor, which makes them worthwhile in integrated crop management systems [29]. Both species exhibited phosphate solubilization, an ideal characteristic to enhance phosphorus availability in soils, a limiting growth nutrient for most plants [30]. However, none of the tested species exhibited nitrogen-fixing activity, suggesting that their PGP effects would rather be mediated through hormone synthesis and nutrient mobilization than atmospheric nitrogen assimilation [10].

Although the tested isolates possessed useful characteristics, the lack of protease, pectinase, and β-1,3-glucanase activity in the three species of *Trichoderma* implies that the antagonistic activities of the three species against fungal pathogens could not be based chiefly on direct cell wall degradation. Rather, their biocontrol activity can be triggered by competition for nutrients, antibiosis (e.g., via production of hydrocyanic acid), or through the induction of systemic resistance in host plants [31]. Failure to produce some of the extracellular enzymes contradicts other reports where *Trichoderma* produced lytic enzymes, pointing towards strain-dependent differences in functional characteristics [32]. Such differences emphasize the necessity of characterization of locally isolated *Trichoderma* strains to identify the best potential candidates for specific biocontrol uses [33]. Overall, PGP variable and enzymatic profiles observed herein affirm *Trichoderma* as a complex bioagent, albeit requiring additional efforts to understand the exact mechanisms governing its pathogen inhibition and plant-beneficial activity under diverse environmental conditions [8,34].

Environmental conditions, especially temperature and pH, largely affected the growth and competitive performance of *Trichoderma* species, as demonstrated in this work. All the three tested *Trichoderma* species exhibited optimum mycelial growth at 30 °C with steep decreases in the rate of growth at the lower (19 °C) and higher (45 °C) ends of the temperature spectrum [35]. This temperature preference agrees with previous reports that most *Trichoderma* species thrive in the moderate temperatures prevailing in agricultural environments [36]. The extreme growth inhibition at 45 °C indicates the possible limitation for agricultural application in high-temperature regions; however, variability within species was evident, with *T. asperellum* being more heat-tolerant than *T. longibrachiatum* and *T. harzianum* [37]. These temperature-sensitive growth responses have basic significance in isolate selection, especially in warmer climates where thermotolerant biocontrol products would be advantageous [38]. The pH tolerance tests also manifested highly notable impacts on fungal growth dynamics. Fungal growth peaked at neutral to slightly alkaline pH (7–9), with *T. asperellum* thriving best, while extreme pH levels hindered growth, especially for *T. longibrachiatum*, highlighting the need for pH-specific species application in agriculture [39].

The environmental sensitivity noted in this study refers to an important factor to be considered for the field application of *Trichoderma*-based biocontrol systems. Though laboratory conditions often reveal spectacular antagonistic potential, performance under field conditions could be significantly affected by changing environmental factors [38,39]. This conclusion is especially true for post-harvest applications where storage conditions could be very variable [36]. Our findings suggest that *T. asperellum* seems to be the most generalist candidate in a variety of conditions, with robust growth under favorable temperature and pH but comparatively good performance under unfavorable conditions. The findings contribute to the growing body of evidence for environmental adaptation matching in selecting microbial biocontrol agents for a particular agricultural application [8]. Future work needs to concentrate on enhancing environmental stress tolerance at the advanced level and on examining possible synergies when a combination of various *Trichoderma* species is used to expand the spectrum of positive environmental conditions [40].

The in vivo assays highlight the increased ability of *Trichoderma* species to repress post-harvest fungal infestations yet uphold and augment tomato fruit quality attributes. *T. longibrachiatum* and *T. asperellum* were severely inhibitory to *A. alternata* and *C. spicifera*, causing reductions in the diameters of the lesions to marked values relative to the pathogen-infected control treatments [41]. Pathogen suppression could be due to more than one mechanism, such as competition for a niche and for nutrients, excretion of antifungal metabolites, and possibly even induction of systemic defense in fruit tissue [8]. The differences revealed among the tested *Trichoderma* species, particularly the comparatively lower performance of *T. harzianum*, may be attributed to isolate-specific variability in functional traits and possibly lower production of antifungal metabolites, which may explain its weaker efficacy. In this context, future studies should focus on characterizing the metabolite profiles of the tested species of *Trichoderma* and improving the environmental adaptability of *T. harzianum* to enhance its biocontrol performance.

In addition to disease control, the *Trichoderma* treatments also had beneficial effects on the tomato fruits’ quality and preserved firmness and chemical balance, showing benefits beyond pathogen control as reported elsewhere [42]. The biochemical assays also provide further information on protection mechanisms, demonstrating that *Trichoderma* treatments significantly reduced malondialdehyde content, a key indicator of oxidative stress compared with pathogen-infected controls [43]. The reduction noted in lipid peroxidation suggests that *Trichoderma* can potentially boost the fruit’s antioxidant defense mechanisms, possibly through the induction of protective enzymes or phenolic compounds. Moreover, the noted increase in total phenolic content in the fruits treated with *T. longibrachiatum* corroborates this assumption because phenolic compounds are renowned for playing critical roles in plant defense and fruit quality preservation [44].

Through controlling pathogens and the quality determinants of fruits, the application of *Trichoderma* can serve to minimize post-harvest loss while satisfying consumers’ demands for produce with better appearance, texture, and nutrition [45].

The results of this study are critically important to formulate sustainable ways to manage tomatoes after harvest and for other uses. The proven efficacy of *Trichoderma* species to control fungal disease without reducing fruit quality provides a strong alternative to traditional chemical fungicides. This helps to address increasing concerns about pesticide residues, contamination, and resistance of pathogens [46,47]. As agriculture across the globe shifts to more sustainable practices, natural allies such as *Trichoderma* provide an effective means to reduce post-harvest losses estimated at 20–50% for perishable produce while maintaining food safety and quality. The efficacy of *T. longibrachiatum*, *T. harzianum*, and *T. asperellum* in this work demonstrates that these species may be integrated into existing post-harvest practices, particularly where *Alternaria* and *Curvularia* infections pose significant risks to tomato storage and marketability [48].

## 5. Conclusions

This work confirmed that *T. longibrachiatum* and *T. asperellum* were effective biocontrol agents against *A. alternata* and *C. spicifera* in post-harvest tomatoes, improving both disease resistance and fruit quality. Their application may reduce post-harvest losses and reliance on chemical fungicides. However, the limit in this work lies in its laboratory-based design, which may not fully reflect commercial storage conditions. Additionally, the screening assays used were appropriate for the comparative evaluation of the *Trichoderma* species, providing preliminary evidence of metabolite production. Therefore, future studies using advanced analytical techniques such as HPLC or LC−MS/MS are needed to quantify the metabolites associated with the observed biological activities. Our current research is focused on validating these findings under large-scale post-harvest systems and evaluating formulation stability, cost-effectiveness, and compatibility with existing storage practices.

## Figures and Tables

**Table 1:** Extracellular enzyme activities and plant-growth-promoting traits of *Trichoderma* spp. (Tr1: *T. longibrachiatum*; Tr2: *T. harzianum*; and Tr3: *T. asperellum*).

*Trichoderma* Species	Cat	Pec	Pro	Amy	Glu	IAA	HCN	N	P
*T. longibrachiatum*	+	−	−	−	−	+	+	−	+
*T. harzianum*	+	−	−	−	−	−	+	−	+
*T. asperellum*	+	−	−	−	−	+	+	−	+

+: enzyme production activity; −: no detectable enzyme production; Cat: catalase; Pec: pectinase, Pro: protease, Amy: amylase, Glu: β-1,3-glucanase; IAA: indole-3-acetic acid; HCN: hydrocyanic acid; N: atmospheric nitrogen; P: phosphate. Five replicates per run, repeated in three independent experiments (n = 5 × 3).

**Table 2:** Mycelial growth (cm) of *Trichoderma* species (*T. longibrachiatum*, *T. harzianum*, and *T. asperellum*) in response to different temperatures during 7 days of incubation (D1, D2, D3, D4, D5, D6, and D7).

Treatments	Temperature (°C)	D1	D2	D3	D4	D5	D6	D7
*T. longibrachiatum*	19	0.07 ± 0.11 b	0.4 ± 0.36 b	2 ± 0.26 b	2.17 ± 0.30 b	2.53 ± 0.55 b	2.9 ± 0.81 b	3 ± 0.79 b
	30	1.7 ± 0.4 a	1.93 ± 0.23 a	2.77 ± 0.50 a	4.77 ± 0.64 a	6.33 ± 0.47 a	7.43 ± 0.41 a	7.63 ± 0.41 a
	45	0 ± 0 b	0 ± 0 b	0.67 ± 0.15 c	1.4 ± 0.50 b	1.9 ± 0.09 b	2.47 ± 0.15 b	2.6 ± 0.1 b
***p*-value**		**<0.01**	**<0.01**	**<0.01**	**<0.01**	**<0.01**	**<0.01**	**<0.01**
*T. harzianum*	19	0.17 ± 0.15 b	1.03 ± 0.11 b	2.57 ± 0.49 ab	3.17 ± 0.05 b	4.03 ± 0.28 b	5.47 ± 0.49 b	5.6 ± 0.51 b
	30	2.7 ± 0.19 a	2.93 ± 0.25 a	3.07 ± 0.20 a	4.43 ± 0.72 a	5.4 ± 0.62 a	6.8 ± 0.26 a	7.27 ± 0.37 a
	45	0 ± 0 b	0 ± 0 c	2.13 ± 0.25 b	2.47 ± 0.20 b	2.97 ± 0.41 c	3.27 ± 0.66 c	3.37 ± 0.75 c
***p*-value**		**<0.01**	**<0.01**	**<0.05**	**<0.01**	**<0.01**	**<0.01**	**<0.01**
*T. asperellum*	19	0.63 ± 0.11 b	1.03 ± 0.15 b	3.43 ± 0.11 a	4 ± 0.17 a	4.33 ± 0.32 b	4.9 ± 0.43 ab	5.13 ± 0.47 b
	30	2.6 ± 0.34 a	3 ± 0.17 a	3.27 ± 0.32 a	4.23 ± 0.51 a	5.67 ± 0.45 a	6.3 ± 0.40 a	6.63 ± 0.25 a
	45	0.1 ± 0.01 c	0.13 ± 0.05 c	0.23 ± 0.11 b	2.03 ± 0.15 b	3.1 ± 0.60 c	4.03 ± 0.96 b	4.17 ± 0.80 b
***p*-value**		**<0.01**	**<0.01**	**<0.01**	**<0.01**	**<0.01**	**<0.05**	**<0.01**

The values represent the mean of the 5 replicates, repeated in three independent experiments (n = 5 × 3), ± standard error. Means with different lowercase letters are significantly different (Duncan’s Multiple Range Test at *p* ≤ 0.05).

**Table 3:** Temporal variation in mycelial growth (cm) of *Trichoderma* species (*T. longibrachiatum*, *T. harzianum*, and *T. asperellum*) in response to different pH during 7 d of incubation (D1, D2, D3, D4, D5, D6, and D7).

Treatments	pH	D1	D2	D3	D4	D5	D6	D7
*T. longibrachiatum*	5	1.23 ± 0.15 a	1.53 ± 0.05 a	1.97 ± 0.11 a	2.47 ± 0.30 a	2.93 ± 0.37 a	4.4 ± 0.7 a	4.63 ± 0.51 a
	7	0.83 ± 0.30 ab	1.17 ± 0.20 ab	1.53 ± 0.05 ab	2.13 ± 0.25 a	4.2 ± 1.30 a	5.13 ± 0.96 a	5.77 ± 0.30 a
	9	0.47 ± 0.05 bc	0.73 ± 0.15 bc	1 ± 0.26 bc	2.9 ± 1.32 a	4.73 ± 2.46 a	8.63 ± 1.30 a	8.7 ± 2.30 a
	11	0.13 ± 0.11 c	0.33 ± 0.28 c	0.47 ± 0.40 c	1.83 ± 0.61 a	3.37 ± 0.66 a	4.93 ± 0.37 a	5.13 ± 0.20 a
***p*-value**		**<0.01**	**<0.01**	**<0.01**	**>0.05**	**>0.05**	**>0.05**	**>0.05**
*T. harzianum*	5	2.77 ± 0.40 a	3.03 ± 0.41 a	3.60 ± 0.36 a	3.87 ± 0.20 a	4.57 ± 0.50 a	5.3 ± 0.09 a	5.37 ± 0.11 a
	7	1.33 ± 0.11 b	1.63 ± 0.20 b	1.93 ± 0.23 b	2.63 ± 0.41 a	3.87 ± 0.35 a	5.6 ± 0.26 a	5.97 ± 0.15 a
	9	0.53 ± 0.32 c	0.77 ± 0.30 c	1.17 ± 0.31 bc	3.63 ± 1.05 a	5.17 ± 1.92 a	6.77 ± 1.77 a	7.1 ± 1.74 a
	11	0.07 ± 0.11 c	0.23 ± 0.25 c	0.47 ± 0.45 c	0.93 ± 0.95 b	2.50 ± 0.52 a	4.13 ± 0.30 a	4.30 ± 0.26 a
***p*-value**		**<0.01**	**<0.01**	**<0.01**	**<0.01**	**>0.05**	**>0.05**	**>0.05**
*T. asperellum*	5	1.90 ± 0.36 a	2.13 ± 0.25 a	2.67 ± 0.28 a	3 ± 0.26 bc	3.73 ± 0.32 b	4.83 ± 0.15 b	5.17 ± 0.11 b
	7	2.97 ± 0.46 a	3.2 ± 0.43 a	3.6 ± 0.36 a	5.33 ± 1.56 a	6.87 ± 1.71 a	8.47 ± 3.19 ab	8.67 ± 2.99 ab
	9	2.43 ± 0.68 a	2.63 ± 0.61 a	3.03 ± 0.41 a	4.53 ± 0.55 ab	7.33 ± 1.53 a	12.2 ± 1.64 a	12.27 ± 1.61 a
	11	0.37 ± 0.35 b	0.50 ± 0.45 b	0.73 ± 0.60 b	1.30 ± 0.62 c	3.07 ± 1.09 b	4.30 ± 0.60 b	4.43 ± 0.49 b
***p*-value**		**<0.01**	**<0.01**	**<0.01**	**<0.01**	**<0.01**	**<0.01**	**<0.01**

The values represent the mean of the 5 replicates, repeated in three independent experiments (n = 5 × 3), ± standard error. Means with different lowercase letters are significantly different (Duncan’s Multiple Range Test at *p* ≤ 0.05).

**Table 4:** Effect of preventive treatments of *Trichoderma* species (Tr1: *T. longibrachiatum*; Tr2: *T. harzianum*; and Tr3: *T. asperellum*) and salicylic acid (SA) on the pH, electrical conductivity, sugar content, titratable acidity, nitrate content, and firmness in tomato fruits inoculated with *Alternaria alternata* and *Curvularia spicifera* under laboratory conditions.

Treatments	pH	Electrical Conductivity (mS/cm)	Sugar Content (°Brix)	Titratable Acidity (g/10 mL)	Nitrate Content (µg/mL)	Firmness
***Alternaria alternata***						
***T. longibrachiatum***	4.24 ± 0.07 bcd	4.43 ± 0.02 d	6.47 ± 0.15 b	7 ± 0.5 c	1633.33 ± 2.08 d	2.43 ± 0.31 bc
***T. harzianum***	4.1 ± 0.01 cd	4.35 ± 0.19 d	6.47 ± 0.06 b	8.1 ± 0.3 a	2466.67 ± 1.15 b	2.07 ± 0.72 c
***T. asperellum***	4.5 ± 0.04 abc	6.17 ± 0.04 a	4.57 ± 0.31 d	3.97 ± 0.15 f	2866.67 ± 1.16 a	2.87 ± 0.25 ab
**Salicylic acid**	4.64 ± 0.26 ab	5.95 ± 0.07 b	5.83 ± 0.12 c	5.33 ± 0.06 d	1500 ± 1 d	3.13 ± 0.25 ab
**C+**	4.71 ± 0.48 a	1.4 ± 0.11 e	6.53 ± 0.06 b	4.7 ± 0.20 e	690 ± 0.2 e	0.5 ± 0.17 d
**C−**	4.06 ± 0.03 d	5.48 ± 0.10 c	7.5 ± 0.10 a	7.53 ± 0.25 b	1966.7 ± 0.6 c	3.43 ± 0.47 a
***p*-value**	**<0.05**	**<0.01**	**<0.01**	**<0.01**	**<0.01**	**<0.01**
***Curvularia spicifera***						
***T. longibrachiatum***	4.4 ± 0.11 a	6.49 ± 0.04 a	4.63 ± 0.15 d	5.63 ± 0.25 c	2400 ± 1 a	2.67 ± 0.25 b
***T. harzianum***	4.17 ± 0.02 b	6.19 ± 0.24 b	6.7 ± 0.09 b	5.2 ± 0.19 d	2466.67 ± 1.1 a	1.43 ± 0.15 c
***T. asperellum***	4.24 ± 0.06 b	2.35 ± 0.20 d	6.4 ± 0.17 c	4.67 ± 0.21 e	2333.33 ± 2.08 a	2.47 ± 0.15 b
**Salicylic acid**	4.16 ± 0.02 b	1.25 ± 0.02 e	6.3 ± 0.10 c	6.67 ± 0.21 b	2266.67 ± 1.15 ab	1.1 ± 0.20 c
**C+**	4.02 ± 0.03 c	1.03 ± 0.02 e	6.47 ± 0.06 c	4.37 ± 0.15 e	2266.67 ± 2.89 ab	0.53 ± 0.47 d
**C−**	4.06 ± 0.04 c	5.48 ± 0.1 c	7.5 ± 0.10 a	7.53 ± 0.25 a	1966.67 ± 0.58 b	3.43 ± 0.47 a
***p*-value**	**<0.01**	**<0.01**	**<0.01**	**<0.01**	**<0.05**	**<0.01**

The value represents the mean 10 replicates per block across three blocks, evaluated in two independent experimental repetitions (n = 10 × 3 ×2), ± standard error. Means with different letters indicate significant differences according to Duncan’s Multiple Range Test (*p* ≤ 0.05.) C+: infected control; C−: negative control.

**Table 5:** Effect of preventive treatments of the tested *Trichoderma* species (Tr1: *T. longibrachiatum*; Tr2: *T. harzianum*; and Tr3: *T. asperellum*) and salicylic acid (SA) on malondialdehyde, total phenolic content, and total protein content in tomato fruits inoculated with *Alternaria alternata* and *Curvularia spicifera* under laboratory conditions.

Treatments	Malondialdehyde (µmol/g)	Total Protein Content (mg/g)	Total Phenolic Content (µg/g)
***Alternaria alternata***			
***T. longibrachiatum***	2.22 ± 0.007 b	26.2 ± 0.01 a	1.11 ± 0.02 a
***T. harzianum***	2.46 ± 0.008 a	19.22 ± 0.01 a	0.84 ± 0.06 bc
***T. asperellum***	1.38 ± 0.005 d	21.32 ± 0.004 a	0.68 ± 0.01 c
**Salicylic acid**	2.12 ± 0.006 bc	22.87 ± 0.02 a	0.71 ± 0.007 c
**Infected control**	2.13 ± 0.003 bc	17.21 ± 0.004 a	0.39 ± 0.006 d
**Negative control**	2.04 ± 0.02 c	14.88 ± 0.05 a	1.04 ± 0.01 ab
***p*-value**	**<0.01**	**≥0.05**	**<0.01**
***Curvularia spicifera***			
***T. longibrachiatum***	1.86 ± 0.01 d	19.46 ± 0.02 b	0.91 ± 0.009 b
***T. harzianum***	2.73 ± 0.006 b	12.71 ± 0.007 b	0.7 ± 0.003 c
***T. asperellum***	2.32 ± 0.006 c	31.24 ± 0.02 ab	0.78 ± 0.01 c
**Salicylic acid**	2.11 ± 0.007 cd	50.85 ± 0.1 a	0.74 ± 0.003 c
**Infected control**	3.32 ± 0.004 a	15.5 ± 0.02 b	0.59 ± 0.01 d
**Negative control**	2.04 ± 0.02 cd	14.88 ± 0.05 b	1.04 ± 0.01 a
***p*-value**	**<0.01**	**<0.01**	**<0.01**

The value represents the mean of 10 replicates per block across three blocks, evaluated in two independent experimental repetitions (n = 10 × 3 × 2), ± standard error. Means with different letters indicate significant differences according to Duncan’s Multiple Range Test (*p* ≤ 0.05.) C+: infected control; C−: negative control.

## Data Availability

The original contributions presented in the study are included in the article/Supplementary Material; further inquiries can be directed to the corresponding authors.

## Supplementary Materials

The following supporting information can be downloaded at https://www.mdpi.com/article/10.3390/jof12060421/s1. Figure S1: Plate confrontation assays. The plate assay to identify the antifungal activities of Tr1: *Trichoderma longibrachiatum*, Tr2: *Trichoderma harzianum*, and Tr3: *Trichoderma asperellum* against the mycelial growth of *Alternaria alternata* and *Curvularia specifera*; Figure S2: Representative photographs of tomato seedlings showing the differences between control and filtrate dilutions at 0, 1/10, 1/50, 1/100, 1/200, and 1/500 of *Trichoderma longibrachiatum* fungal application; Figure S3: Photographs showing the effects of *Trichoderma longibrachiatum*, *T. harzianum*, *T. asperellum*, and salicylic acid treatments on tomato fruits inoculated with *Alternaria alternata* and *Curvularia spicifera*.
