# Supplementary material for: Estimating Efficacy of Indigenous Isolates of Three Trichoderma Species as Biocontrol Agents Against Alternaria alternata and Curvularia spicifera
Source: J Fungi (Basel). 2026 Jun 10;12(6):421. doi: 10.3390/jof12060421 (PMC13302021; doi:10.3390/jof12060421)
Supplement: Supplementary file 1 [file jof-12-00421-s001.zip › jof-4269575-supplementary.pdf]

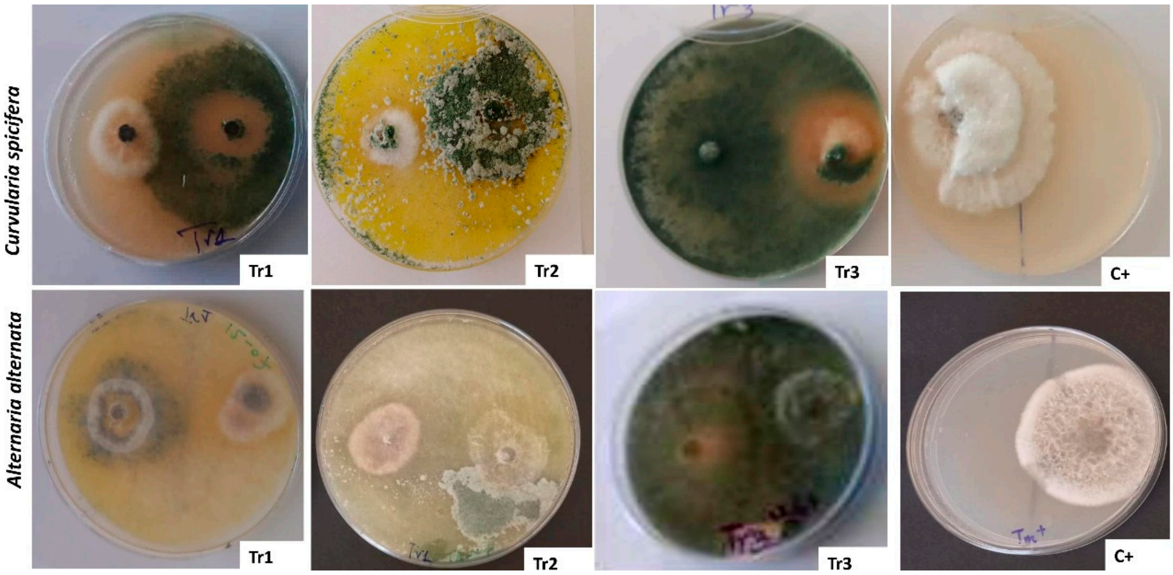

Figure S1. Plate confrontation assays. The plate assay to identify the antifungal activities of Tr1: *Trichoderma longibrachiatum*, Tr2: *Trichoderma harzianum*, and Tr3: *Trichoderma asperellum* against the mycelial growth of *Alternaria alternata* and *Curvularia specifera*.

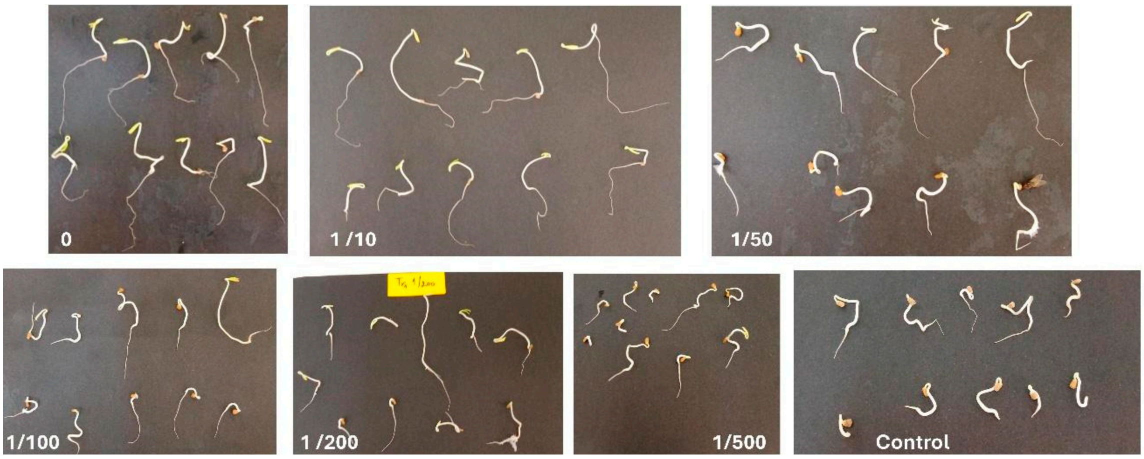

Figure S2. Representative photographs of tomato seedlings showing the differences between control and filtrate dilutions at 0, 1/10, 1/50, 1/100, 1/200, and 1/500 of *Trichoderma longibrachiatum* fungal application.

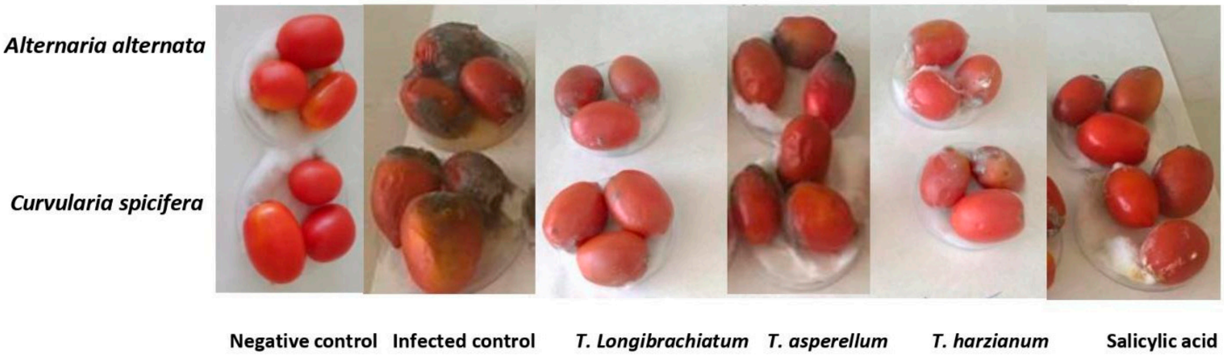

Figure S3. Photographs showing the effects of *Trichoderma longibrachiatum, T. harzianum, T. asperellum,* and salicylic acid treatments on tomato fruits inoculated with *Alternaria alternata* and *Curvularia spicifera*.
